# Validation of accuracy in image co-registration with computed tomography and magnetic resonance imaging in Gamma Knife radiosurgery

**DOI:** 10.1093/jrr/rru027

**Published:** 2014-04-29

**Authors:** Hisato Nakazawa, Yoshimasa Mori, Masataka Komori, Yuta Shibamoto, Takahiko Tsugawa, Tatsuya Kobayashi, Chisa Hashizume

**Affiliations:** 1 Department of Radiological Sciences, Nagoya University Graduate School of Medicine, Nagoya, Aichi, Japan; 2 Nagoya Radiosurgery Center, Nagoya Kyoritsu Hospital, Nagoya, Aichi, Japan; 3 Department of Radiology and Radiation Oncology, Aichi Medical University, Nagakute, Aichi, Japan; 4 Department of Radiology, Nagoya City University Graduate School of Medical Sciences, Nagoya, Aichi, Japan

**Keywords:** magnetic resonance imaging, computed tomography, pre-planning, co-registration, stereotactic radiosurgery

## Abstract

The latest version of Leksell GammaPlan (LGP) is equipped with Digital Imaging and Communication in Medicine (DICOM) image-processing functions including image co-registration. Diagnostic magnetic resonance imaging (MRI) taken prior to Gamma Knife treatment is available for virtual treatment pre-planning. On the treatment day, actual dose planning is completed on stereotactic MRI or computed tomography (CT) (with a frame) after co-registration with the diagnostic MRI and in association with the virtual dose distributions. This study assesses the accuracy of image co-registration in a phantom study and evaluates its usefulness in clinical cases. Images of three kinds of phantoms and 11 patients are evaluated. In the phantom study, co-registration errors of the 3D coordinates were measured in overall stereotactic space and compared between stereotactic CT and diagnostic CT, stereotactic MRI and diagnostic MRI, stereotactic CT and diagnostic MRI, and stereotactic MRI and diagnostic MRI co-registered with stereotactic CT. In the clinical study, target contours were compared between stereotactic MRI and diagnostic MRI co-registered with stereotactic CT. The mean errors of coordinates between images were < 1 mm in all measurement areas in both the phantom and clinical patient studies. The co-registration function implemented in LGP has sufficient geometrical accuracy to assure appropriate dose planning in clinical use.

## INTRODUCTION

Planning of Gamma Knife (GK) stereotactic radiosurgery (SRS) depends on stereotactic images with the fiducial system of the Leksell stereotactic G skull frame (ELEKTA, Tokyo), including computed tomography (CT) and magnetic resonance imaging (MRI). In dealing with brain tumors, MRI is more useful than CT, because MRI visualizes both normal and pathological anatomical structures more clearly. However, attention has to be paid to image distortion caused by magnetic field inhomogeneities induced by the metallic stereotactic skull frame, posts and fixation screws. We previously confirmed that these stereotactic localization errors are acceptably small in the entire stereotactic space using phantoms mounted on a frame [1]. Some authors have also reported that the geometric accuracy of coordinates between MRI and CT images was within tolerance levels by measurement of both phantoms [2] and clinical cases [3–4]. Recently, an automatic image co-registration function has been implemented in Leksell GammaPlan (LGP) Version 10.1.1 for GK treatment planning. This latest version also enables virtual treatment planning with diagnostic images taken without a skull frame prior to GK SRS. The diagnostic images in association with virtual dose distributions can be co-registered on stereotactic images taken with a skull frame on, and then real dose calculations based on the stereotactic coordinate system can be performed. In this study, the accuracy of co-registration was evaluated in a phantom study and in clinical cases to demonstrate the usefulness of this system for pre-planning of diagnostic MRIs.

## MATERIALS AND METHODS

In the phantom study, three kinds of phantoms were used to assess the geometrical accuracy of co-registration between images. First, stereotactic CT and frameless diagnostic CT co-registered with stereotactic CT were compared to evaluate the accuracy of co-registration itself. Second, stereotactic MRI and frameless diagnostic MRI co-registered with stereotactic MRI were compared to evaluate the accuracy including possible image distortion of stereotactic MRI. Third, stereotactic CT and diagnostic MRI co-registered with stereotactic CT were compared to evaluate the accuracy including differences of imaging modalities. Fourth, stereotactic MRI and frameless diagnostic MRI co-registered with stereotactic CT with a skull frame attachment were compared. In addition, clinical investigations in acoustic schwannoma cases were performed to compare between stereotactic MRI and frameless diagnostic MRI co-registered with stereotactic CT with a skull frame attachment. In clinical cases, target delineation and definition is generally performed with MRI, because the tumor delineation is impossible on CT images that have less tissue contrast than MRI. Therefore, it is important to verify the spatial uncertainty between stereotactic MRI and diagnostic MRI co-registered with stereotactic CT.

### Phantoms

**Fig. 1.**
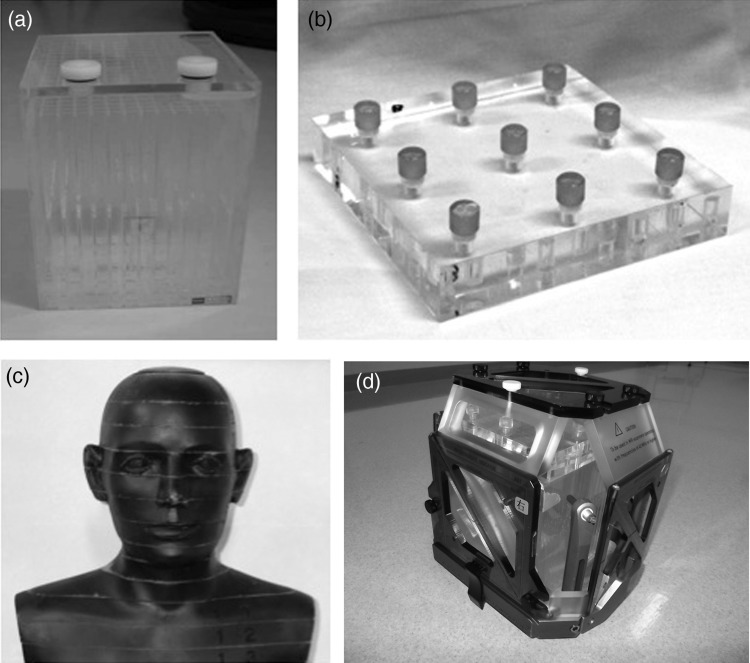
Phantom A: cubic phantom including matrix structures (**a**). Phantom B: acrylic plate phantom with nine small cylinder-shaped baths (**b**). Phantom C: anthropomorphic phantom (**c**). Phantom B firmly fixed on Phantom A is mounted on a stereotactic skull frame with four aluminum posts by fixation screws through small single-use plastic insulator parts (Elekta, Tokyo). Stereotactic fiducial indicator boxes (Elekta, Tokyo) were attached on the frame to provide a stereotactic coordinate system during scanning of stereotactic CT and MRI (**d**).

Three types of phantoms were used: a grid-pattern acrylic box phantom (Phantom A, Fig. 1a), an acrylic plate with nine cylinder-shaped baths (Phantom B, Fig. 1b), and an anthropomorphic phantom simulating human head structures (Phantom C, Fig. 1c). Phantom A consists of a matrix-shaped acrylic structure inside a cubic-shaped box 150 × 150 × 150 mm in size, which is approximately the same volume as the average human adult head. Each cylinder of Phantom B is 8 mm in diameter and 10 mm in height. The inside of the cubic Phantom A and the baths of Phantom B were filled with copper sulfate solution (CuSO_4_, 0.002M). Phantom B was firmly fixed on Phantom A when in use. The phantoms were mounted on a stereotactic skull frame with four aluminum posts by fixation screws through small single-use plastic insulator parts (Elekta, Tokyo). Stereotactic fiducial indicator boxes (Elekta, Tokyo) were attached on the frame to provide a stereotactic coordinate system during scanning of stereotactic CT and MRI (Fig. 1d). The MRI fiducial box is equipped with tubes filled with CuSO_4_ solution, and the CT fiducial box has low-artifact thin copper plates as the fiducial reference.

### Clinical cases

A total of 11 patients with acoustic schwannoma were enrolled. All patients underwent GK SRS between January 2011 and October 2012. As diagnostic MRI, 3-dimensional spoiled gradient recalled acquisition in the steady state (3D-SPGR) images with gadolinium (Gd, 0.1 mmol/kg body weight) contrast enhancement of 1-mm slice thickness were acquired using an 8-channel head coil in a 1.5-Tesla (T) MRI scanner, prior to GK SRS. Virtual dose planning for GK SRS for each acoustic schwannoma was made on the diagnostic MRI. On the day of treatment, stereotactic CT and MRI were performed after attachment of a stereotactic skull frame. Planning on stereotactic CT co-registered with the diagnostic MRI was completed. Independently, real dose planning on stereotactic MRI was performed using stereotactic MRI. Plannings were compared with each other. Contrast-enhanced 3D-SPGR at 1-mm slice thickness was taken using a quadrature head coil in the same scanner. The same dose of Gd was injected after the frame placement for GK SRS. The interval between the pre-procedural diagnostic MRI and the stereotactic MRI on the day of treatment ranged from 1–11 d (mean, 4 d; median, 2 d).

### Diagnostic and stereotactic CT and MRI

A 4-detector CT scanner (Light Speed Plus, GE Healthcare, Tokyo) and a 1.5-T MRI (Echo Speed Plus 1.5 T, GE Healthcare, Tokyo) with two types of radiofrequency coil (8-channel head coil and Quadrature head coil; GE Healthcare, Tokyo), with peak amplitude of gradient of 33 mT/m and a peak slew rate of 120 T/m/s were used. 3D-SPGR was applied as a pulse sequence. Details of the acquisition parameters of 3D-SPGR MRI and CT are shown in Table 1. In the clinical patient study, axial slices contiguous to the skull frame plane were taken in the phantom study and during stereotactic 3D-SPGR MRI and stereotactic CT. In the pre-procedural diagnostic MRI for clinical cases, axial slices for 3D-SPGR were applied to the orbitomeatal baseline of the head. The orientations of the axes in the stereotactic space coordinates system were *x* as right–left direction, *y* as posterior–anterior direction, and *z* as superior–inferior direction. The slice thickness of images was 1.25 mm and 1.0 mm without a gap, and the pixel size was 0.5 mm (*x*) by 0.5 mm (*y*) and 1.0 mm (*x*) by 1.0 mm (*y*) in CT and in 1.5 T-MRI, respectively.

**Table 1.** Detailed sequence parameters and sequence order for both diagnostic and stereotactic 3D-SPGR and stereotactic CT scan

	3D-SPGR			CT
	Diagnosis	GK surgery		
Coil	8-channel	Quadrature		
Plane	Axial	Axial	Scan mode	Non-helical
Mode	3D	3D	Voltage (kV)	120
TE (ms)	Min Full (8)	Min Full (5.06)	Current (mA)	170
TR (ms)	30	33	Matrix size	512 × 512
ETL	1	1	Number of slices	120
Flip angle (°)	30	30	Slice thickness (mm)	1
Bandwidth (kHz)	15.63	15.63	Slice gap (mm)	0
NEX	0.75	1	FOV (cm)	30
FOV (cm)	24	24		
Matrix size	256 × 256	256 × 256		
Slice thickness (mm)	1	1		
No. of slices	60	60		
Scan time (min)	6:12	9:10		
Imaging options	ZIP2, FC	ZIP2, FC		

TE = echo time, TR = repetition time, ETL = echo train length, NEX = number of excitations, FOV = field of view, ZIP = zero full interpolation, SPGR = spoiled gradient recalled acquisition in the steady state, GK = Gamma Knife.

Diagnostic CT and MRI without a frame and stereotactic CT and MRI with a stereotactic frame were acquired in the phantom study. Meanwhile, in the clinical study, only pre-operative MRI and stereotactic CT and MRI for dose planning were executed. Only the stereotactic images among the images loaded in LGP were registered with the stereotactic coordinates system by fiducial markers on the localizer box. Diagnostic images were co-registered to the stereotactic images by using mutual information (MI) based on anatomical structures between two image sets.

### Evaluation of image co-registration accuracy

**Fig. 2.**
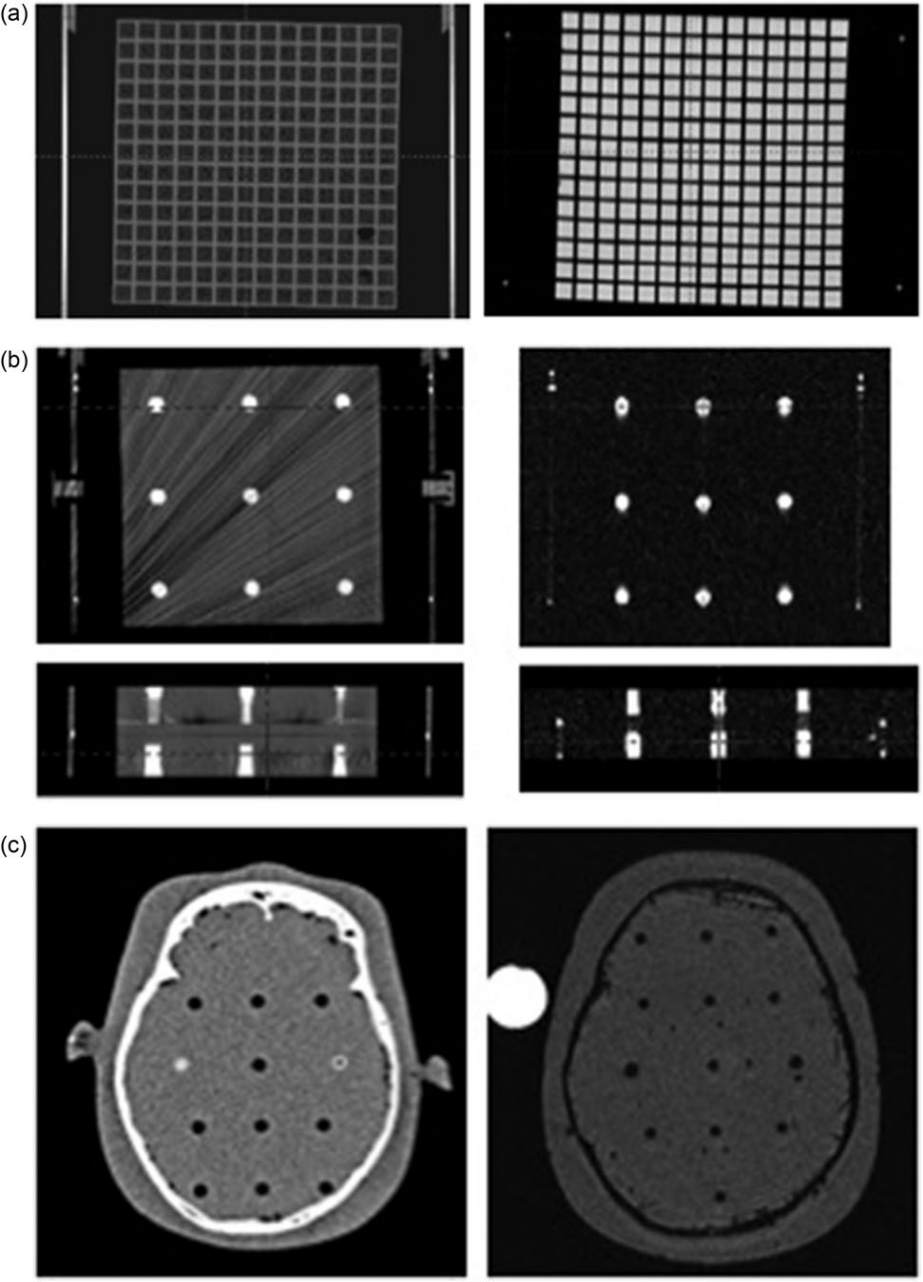
Axial images of Phantom A including matrix structures on CT (left) and MRI (right) (**a**), axial (upper) and coronal (lower) images of Phantom B with nine small cylinder-shaped baths on CT (left) and MRI (right) (**b**), and axial images of Phantom C on CT (left) and MRI (right) (**c**).

**Fig. 3.**
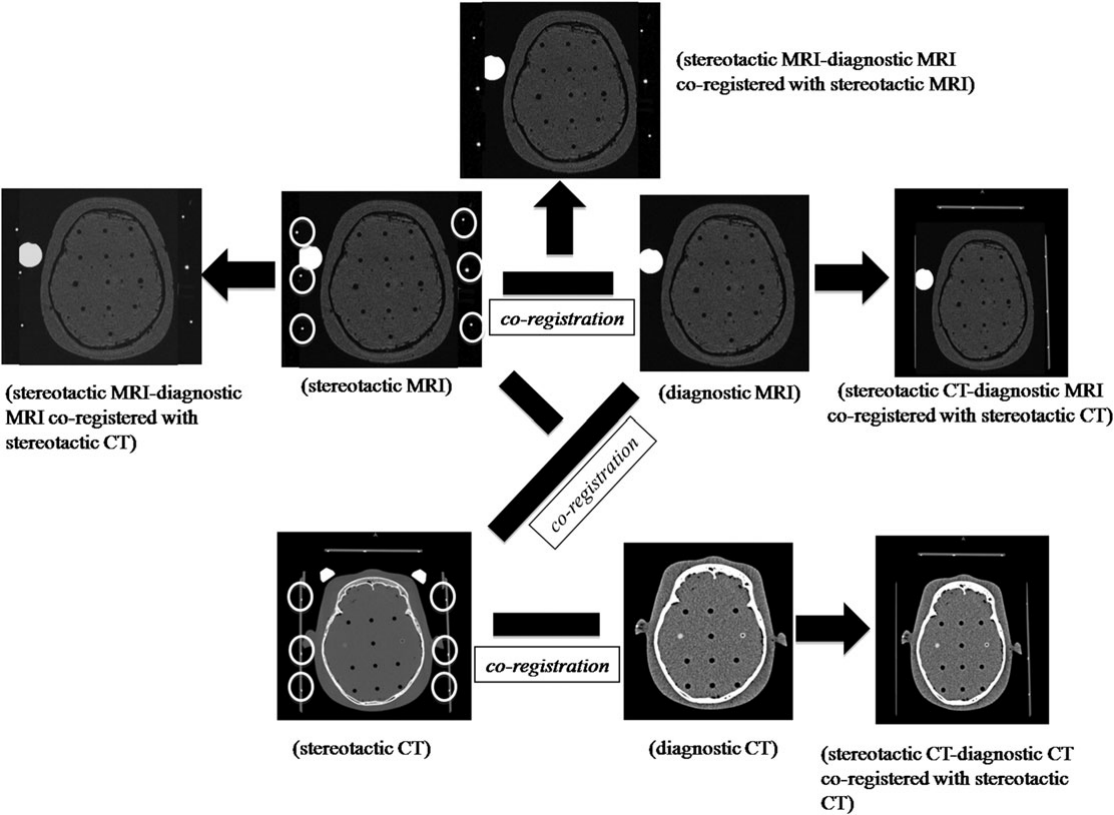
Illustration of four-pair image comparison among stereotactic CT and diagnostic CT co-registered with stereotactic CT; stereotactic MRI and diagnostic MRI co-registered with stereotactic MRI; stereotactic CT and diagnostic MRI co-registered with stereotactic CT; and stereotactic MRI and diagnostic MRI co-registered with stereotactic CT. Here, ‘stereotactic’ means that the image includes the coordinate system by defining fiducial markers. ‘Co-registration’ on the Leksell GammaPlan treatment-planning system is performed by combination of stereotactic CT and diagnostic CT, by combination of stereotactic MRI and diagnostic MRI, and by combination of stereotactic CT and diagnostic MRI.

In the phantom study, some of the authors, at least one neurosurgeon and one radiation oncologist, separately measured the coordinates by the submillimeter. Measurement of the coordinates was performed three times for each of the measuring points. The coordinates of the crossing points in the matrix structure of Phantom A (Fig. 2a) and those of the center points of circles in Phantom C (Fig. 2c) (around *z* = 50, 75, 100, 125, 150) were measured for *x*- and *y*-dimensions (around *x* = 50, 100, 150 and *y* = 50, 100, 150) on different axial planes (*z* = 50, 75, 100, 125, 150) to evaluate errors of coordinates between image sets, including stereotactic CT and diagnostic CT co-registered with stereotactic CT, stereotactic MRI and diagnostic MRI co-registered with stereotactic MRI, stereotactic CT and diagnostic MRI co-registered with stereotactic CT, and stereotactic MRI and diagnostic MRI co-registered on stereotactic CT with the skull frame attachment (Fig. 3). For the *z*-dimension, the coordinates of each center of the cylinder-shaped baths of Phantom B were measured (around *z* = 50, 75, 100, 125, 150 at around *x* = 50, 100, 150, and *y* = 50, 100, 150) (Fig. 2b).

In the clinical study, the difference between the target contour on the actual stereotactic MRI and that on the pre-planning diagnostic MRI co-registered with the stereotactic CT was evaluated in 11 cases of acoustic schwannoma. Pre-procedural diagnostic MRI without frame attachment was co-registered in stereotactic CT with a frame on the patient's head taken on the day of treatment. The target contour was delineated in a manner consistent with clinical practice both on stereotactic and diagnostic 3D-SPGR MRIs with Gd contrast enhancement. At least one neurosurgeon and one radiation oncologist verified the contours, which were made by one of the authors, a radiation therapy technologist with extensive experience in GK SRS. The window levels and window widths were adjusted to a certain contrast level and kept constant while maintaining the same conditions for all contouring tasks. Both contours defined as targets were exported to a workstation running MIM Maestro Version 5.6.1 (MIM Software, Tokyo) in order to compare the difference of the gravity center coordinates of the target contours. In addition, the volumes of both targets were compared.

## RESULTS

**Fig. 4.**
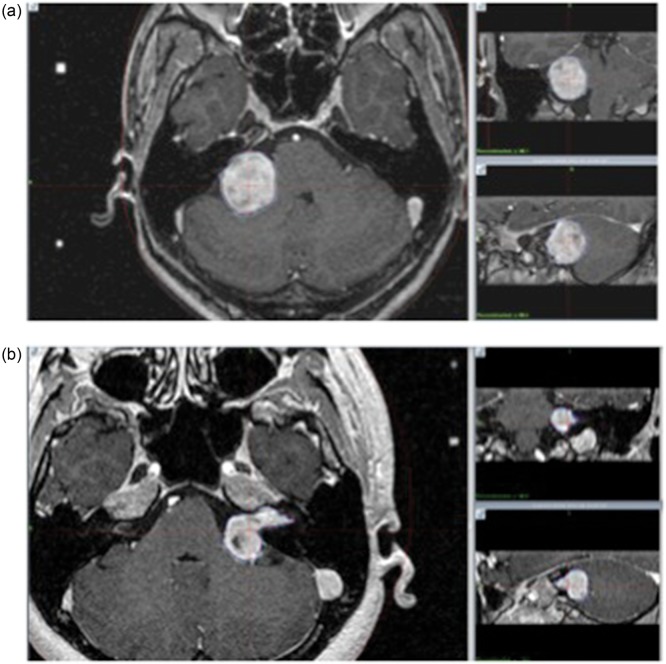
Target contours of Case 1 (**a**) and Case 10 (**b**) of vestibular schwannoma in three planes (axial, coronal and sagittal). In both cases, the delineation on stereotactic MRI (purple) and that on diagnostic MRI co-registered with stereotactic CT (blue) show good agreement.

Tables 2, 3, 4 and 5 show the maximum relative errors of coordinates of the three phantoms between stereotactic CT and diagnostic CT co-registered with stereotactic CT, stereotactic MRI and diagnostic MRI co-registered with stereotactic MRI, stereotactic CT and diagnostic MRI co-registered with stereotactic CT, and stereotactic MRI and diagnostic MRI co-registered with stereotactic CT, respectively, in three different phantoms. There were no significant differences in the *x*, *y* and *z* locations over the entire area of stereotactic space regardless of the *z* position among direct-registered images, including between stereotactic CT and diagnostic CT, between stereotactic MRI and diagnostic MRI, and between stereotactic CT and diagnostic MRI. All measurements were within 1 mm. The errors between stereotactic MRI and diagnostic MRI co-registered with stereotactic CT for indirectly co-registered stereotactic CT were larger than those between the remaining three pairs. The maximum errors between stereotactic MRI and diagnostic MRI co-registered with stereotactic CT in the *x*, *y* and *z* dimensions were 0.9, 1.4 and 1.6 mm for Phantoms A and B respectively, and were 1, 1.5 mm and N/A for Phantom C. Table 6 summarizes the target volumes and the differences in the coordinates of the target centers on stereotactic MRI and diagnostic MRI co-registered with stereotactic CT in 11 cases of acoustic schwannoma. The target volume ranged from 0.64–10.91 ml on stereotactic MRI and from 0.61 to 10.7 ml on pre-planning MRI. The maximum difference was 0.21 ml in Case 1. The maximum differences of coordinates of the center among all cases were 0.7, 1.59, 1.4 and 1.66 mm in the *x*, *y*, *z* dimensions and RMS (root mean square, combined three direction errors on each plane), respectively. The averages of errors in the *x*, *y*, *z* dimensions and RMS errors were 0.32 ± 0.23 mm (mean ± 1σ), 0.71 ± 0.38 mm, 0.73 ± 0.4 mm and 1.06 ± 0.6 mm, respectively. Most cases were less than 1 mm in each dimension. Figure 4 presents demonstrative cases of target volumes, independently delineated on stereotactic MRI and diagnostic MRI. These images indicated good correspondence of the target contours with each other.

**Table 2.** Maximum relative error (mm) of coordinates among nine target points in each dimension on each *z*-plane, compared with stereotactic CT and diagnostic CT co-registered with stereotactic CT

	Phantom A		Phantom B	Phantom C	
z-position	X	Y	Z	X	Y
50	0.5	0.3	0.3	0.7	0.6
75	0.2	0.4	0.1	0.3	0.5
100	0.4	0.3	0.3	0.4	0.8
125	0.5	0.4	0.3	0.3	0.6
150	0.5	0.4	0.2	0.8	0.8

**Table 3.** Maximum relative error (mm) of coordinates among nine target points in each dimension on each *z*-plane, compared with stereotactic MRI and diagnostic MRI co-registered with stereotactic MRI

	Phantom A		Phantom B	Phantom C	
z-position	X	Y	Z	X	Y
50	0.5	0.8	0.8	0.6	1.0
75	0.4	0.6	0.5	0.5	0.5
100	0.3	0.5	0.3	0.4	0.6
125	0.4	0.7	0.7	0.6	0.8
150	0.6	0.8	0.8	0.6	0.8

**Table 4.** Maximum relative error (mm) of coordinates among nine target points in each dimension on each *z*-plane, compared with stereotactic CT and diagnostic MRI co-registered with stereotactic CT

	Phantom A		Phantom B	Phantom C	
*z*-position	X	Y	Z	X	Y
50	0.5	0.5	0.5	0.8	0.8
75	0.5	0.2	0.3	0.4	0.5
100	0.5	0.3	0.9	0.4	0.9
125	0.3	0.4	0.3	0.7	0.5
150	0.4	0.5	0.7	0.9	0.7

**Table 5.** Maximum relative error (mm) of coordinates among nine target points in each dimension on each *z*-plane, compared with stereotactic MRI and diagnostic MRI co-registered with stereotactic CT with the frame

	Phantom A		Phantom B	Phantom C	
z-position	X	Y	Z	X	Y
50	0.7	1.4	1.5	0.9	1.5
75	0.5	1.0	1.0	0.7	1.3
100	0.5	0.9	1.1	0.8	1.1
125	0.9	1.1	1.2	0.9	1.5
150	0.7	1.1	1.6	1.0	1.5

**Table 6.** Summary of the difference in target volume and center of gravity between stereotactic MRI and diagnostic MRI co-registered with stereotactic CT in 11 cases of acoustic schwannoma

Case number	Number of shots	Pre-volume (ml)	GK-volume (ml)	Volume difference (ml)	X (mm)	Y (mm)	Z (mm)	RMS difference (mm)
1	25	10.7	10.91	0.21	0.45	1.10	0.10	1.19
2	6	0.63	0.64	0.01	0.00	1.59	0.50	1.67
3	9	0.61	0.66	0.05	0.60	0.61	1.00	1.32
4	10	1.82	1.80	0.02	0.00	0.79	1.40	1.61
5	8	0.68	0.69	0.01	0.10	0.33	0.80	0.87
6	13	1.81	1.76	0.05	0.25	0.39	0.40	0.61
7	30	6.35	6.27	0.08	0.20	0.73	0.70	1.03
8	20	2.43	2.46	0.03	0.50	0.38	0.60	0.87
9	9	0.80	0.77	0.03	0.40	0.79	1.40	1.66
10	20	2.79	2.79	0	0.30	0.31	0.50	0.66
11	18	2.91	2.84	0.07	0.70	0.74	0.63	1.20
								
mean	15.27	2.87	2.87	0.05	0.32	0.71	0.73	1.06
S.D.	7.84	3.08	3.13	0.06	0.23	0.38	0.40	0.60

RMS = root mean square, S.D. = standard deviation, Pre = diagnostic MRI without the frame, GK = treatment planning MRI with the frame.

## DISCUSSION

Pre-planning MRI may offer many advantages for GK SRS. First, pre-planning allows leisurely pre-procedural planning. It would not be necessary to care for patients with a skull frame on their head waiting for treatment during planning. Second, it may improve the treatment workflow and enhance throughput. If pre-planning has already been completed in advance, only stereotactic CT scanning is necessary after the skull frame attachment. In addition, it takes less time to conduct final treatment planning, with only an adaptive process from the pre-plan being required.

Diagnostic MRI for pre-planning is free from the limitations associated with a skull frame attachment. As the skull frame and the fiducial indicator box may make contact with the surface coil, we are limited to using smaller surface coils during MRI. Our MRI is equipped with both an 8-channel multicoil and a quadrature coil, but the 8-channnel multicoil, which has higher sensitivity and a better signal-to-noise ratio than the quadrature coil, is not available for stereotactic MRI.

**Fig. 5.**
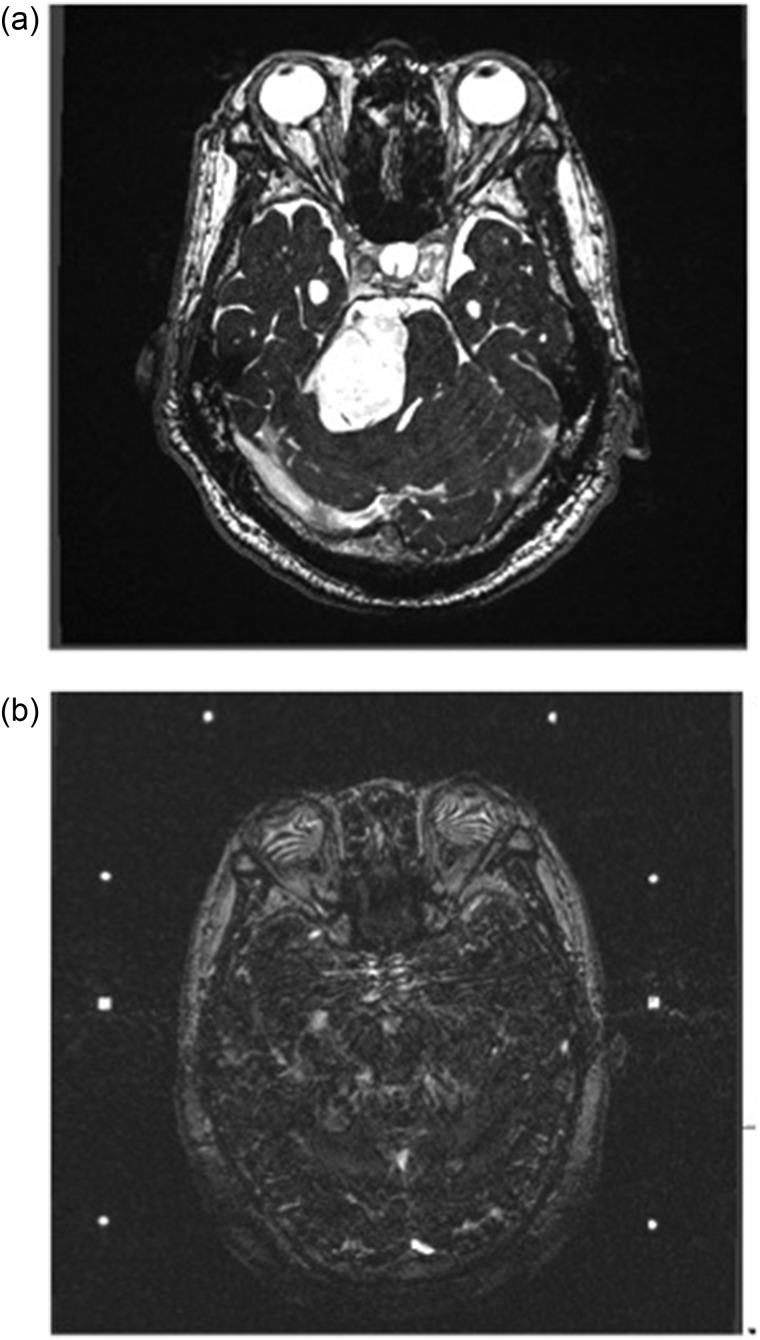
Contrast-enhanced axial FIESTA images of acoustic schwannoma acquired under the same scan conditions with and without a skull frame. Axial image of diagnostic frameless acquisition (**a**) and axial image of stereotactic frame-fixed acquisition (**b**). The metallic frame, posts and screws caused severe susceptibility artifacts (b).

Furthermore, stereotactic MRI may be affected by susceptibility artifacts and distortion caused by the metal skull frame, posts and fixation screws. Fast imaging employing steady state acquisition (FIESTA) serves to improve the contrast. It provides more precise imaging around the skull base [5]. Figure 5 shows contrast-enhanced FIESTA images of an acoustic schwannoma acquired under the same scan conditions with a skull frame (Fig. 5a) and without a frame (Fig. 5b). Figure 5b shows severe artifacts caused by the posts or screws on the FIESTA images. Even if susceptibility artifacts caused by the frame, post and screw are not recognized, image distortion in stereotactic MRI needs to be considered. Nevertheless, because there is no suspicious additional material that may act towards inhomogeneity of the magnetic field, the absolute or geometric position of the diagnostic image without a frame attachment is thought to offer higher reliability and image quality.

Another valuable usage of co-registration function emerged in the follow-up of patients [4]. By co-registration of the subsequent follow-up images with treatment images associated with treatment dose distribution, changes of the lesions could be clarified visually. Besides MRI, positron emission tomography (PET) and single photon emission computed tomography (SPECT) can be co-registered, helping us to assess the viability of the tumor and differentiating tumor recurrence from radiation necrosis [6–7].

This study indicated, both in the phantom study and in the clinical case study, that the accuracy of co-registration by LGP is reliable with an acceptable small error of dimensions. Except for the combination of stereotactic MRI and diagnostic MRI co-registered with stereotactic CT, the accuracy of image co-registration was high, with maximum errors < 1.0 mm. Probably, this is because both image distortion of frame-fixed stereotactic MRI and imperfect co-registration between different modalities of diagnostic MRI and stereotactic CT affect the result in similar directions. In the clinical case study, acoustic schwannoma, being located adjacent to the internal acoustic meatus, is composed of high magnetic field susceptibility factors such as bone and air cavity. These factors have an effect on image distortion. Nevertheless, the errors were not large. In both the phantom and clinical studies, the *y* error (phase-encoding direction) and *z* error (depending on slice thickness) were larger than the *x* error (frequency-encoding direction). The *y* axis was set to the phase direction. Because it takes longer time to fill k-space with MR signals in the phase direction compared with the frequency direction, image is easily affected by distortion. Regarding the *z* error, the resolution of the *z* axis was twice as much as that of the *x* and *y* axes.

In the LGP system, normalized mutual information (NMI) is adopted for the image superposition algorithm [8]. NMI is superior to standard MI [9, 10]. Standard MI only considers similarities of voxels on images but NMI is independent of overlapping of entropies involved when combining two images. NMI works well when images are of different modalities such as MRI and CT or MRI and PET. Veninga *et al*. [11] suggested that the registration accuracy of the NMI-based method might be attributable to the voxel size of the image data, and increased image resolution will necessary to improve the accuracy.

There have been a few phantom and clinical studies for validation of the NMI method. Watanabe *et al*. [12] evaluated the co-registration accuracy of LGP in terms of translation and rotation with an original tower phantom. Using a set of frame and steel markers, Cernica *et al*. [13] also investigated image registration errors resulting from misalignment by rotation of up to 20° in three different planes (axial, coronal and sagittal) around the image axis. The outcomes of their phantom test showed accurate performance. Similarly, in a clinical case study, Bond *et al*. [14] evaluated the NMI method in staged radiosurgery of nine patients with large arteriovenous malformations. Massager *et al*. [4] also investigated the targeting accuracy for trigeminal neuralgia between planning MRI and post-treatment follow-up MRI using an old version of the LGP Multi View frameless co-registration software. As a factor common to both studies, they were limited to only MRI–MRI co-registration. Furthermore, in the latter group, the mean deviation of the target coordinates gained from 65 patients was similar to our results; however the interval of MRI acquisition was significantly different (this study: maximum, 11 days; Massager *et al*.: 6 months). Because of the longer period, they considered that changes in the brain or Vth nerve might have caused position deviations.

There are various possible approaches for multi-modality medical image registration [8–10, 15]; each registration technique has advantages and disadvantages. Recently, the MI [9]-based and NMI [8]-based registration methods have been implemented in commercially available treatment-planning software. There have been few reports of validation of image registration with submillimeter precision (as is required in GK) so far. We found that the co-registration error was < 1.0 mm both in the phantom and the clinical study.

## CONCLUSION

We investigated the accuracy of image co-registration available in an LGP workstation in both a phantom study and a clinical patient study. The geometrical accuracy of the diagnostic MRI co-registered with stereotactic CT is valid for clinical use. In some cases, the stereotactic MRI in certain sequences included more distortion (caused by the metal frame) than the diagnostic MRI co-registered with stereotactic CT. The spatial uncertainty of image co-registration might exceed the level of 1 mm between stereotactic and diagnostic MRI, because the inhomogeneous magnetic field caused geometric distortion. In such cases, treatment planning performed on stereotactic CT and diagnostic MRI (without a metal frame) co-registered on it might be better, because it has less distortion.
